# The value of small airway function parameters and fractional exhaled nitric oxide for predicting positive methacholine challenge test in asthmatics of different ages with FEV_1_ ≥ 80% predicted

**DOI:** 10.1002/clt2.12007

**Published:** 2021-03-23

**Authors:** Lili Hou, Huijuan Hao, Gang Huang, Jinkai Liu, Li Yu, Lei Zhu, Huahao Shen, Min Zhang

**Affiliations:** ^1^ Department of Respiratory and Critical Care Medicine Shanghai General Hospital Shanghai Jiao Tong University School of Medicine Shanghai China; ^2^ Key Laboratory of Respiratory Disease of Zhejiang Province Department of Respiratory and Critical Care Medicine Second Affiliated Hospital of Zhejiang University School of Medicine Hangzhou Zhejiang China; ^3^ Department of Pulmonary and Critical Care Medicine Tongji Hospital Tongji University School of Medicine Shanghai China; ^4^ Department of Respiratory and Critical Care Medicine Zhongshan Hospital Affiliated to Fudan University Shanghai China

**Keywords:** age, asthma, fractional exhaled nitric oxide, methacholine challenge test, small airway, 关键词: 年龄, 哮喘, 呼出气一氧化氮, 乙酰胆碱激发试验, 小气道

## Abstract

**Background:**

Small airway function parameters (SAFPs) combined with fractional exhaled nitric oxide (FeNO) can predict a positive methacholine challenge test (MCT) for asthma diagnosis. However, their predictive utility in patients with forced expiratory volume in one second (FEV_1_) ≥80% predicted within different age ranges remains unclear. This study aimed to assess the utility of SAFPs, alone or combined with FeNO, to predict a positive MCT in patients in two age groups (<55 and ≥55 years) with asthma‐suggestive symptoms and FEV_1_ ≥80% predicted.

**Methods:**

We enrolled 846 Chinese patients with suspected asthma and standard spirometry, FeNO, and MCT findings. Using the area under the curves (AUCs), the utility of SAFPs, alone or combined with FeNO, for predicting a positive MCT was analyzed in a discovery (*n* = 534) and validation cohort (*n* = 312) in both age groups with FEV_1_ ≥80% predicted.

**Results:**

In the discovery cohort, the optimal cut‐off values for predicting a positive MCT in patients aged <55 years (74.2% and 74.9% for forced expiratory flow (FEF)_50%_ and FEF_25%–75%_, respectively) were higher than those in patients aged ≥55 years (65.0% and 62.9% for FEF_50%_, FEF_25%–75%_, respectively). However, the optimal FeNO value in patients aged <55 years (43 ppb) was lower than that in patients aged ≥55 years (48 ppb). FeNO combined with SAFPs (FEF_50%_, FEF_25%–75%_) significantly increased the AUCs in both groups (≥55 years [0.851 for FEF_50%_ and 0.844 for FEF_25%–75%_]; <55 years [0.865 for FEF_50%_ and 0.883 for FEF_25%–75%_]) compared with a single parameter (*p* < 0.05). These findings were confirmed in the validation cohort. Compared with patients ≥55 years, those aged <55 years had higher and lower optimal cut‐off values for SAFPs and FeNO, respectively. The AUCs of FeNO combined with SAFPs for predicting a positive MCT for asthma diagnosis were significantly higher than those of the individual parameters (*p* < 0.05) in both age groups.

**Conclusions:**

There were age‐group differences in the utility of SAFPs combined with FeNO for predicting a positive MCT. Patients with an asthma‐suggestive history and a normal FEV_1_ should be stratified by age when using SAFPs combined with FeNO to predict a positive MCT.

## 1 BACKGROUND

Asthma is a common disorder caused by chronic inflammation of the lower respiratory tract. Its diagnosis is based on typical symptoms of wheeze, recurrent breath shortness, chest tightness, and cough, as well as evidence of variable expiratory airflow limitation based on objective pulmonary function tests.^1^ Both a bronchodilating test with salbutamol and a methacholine challenge test (MCT) are used for asthma diagnosis in clinical practice.^2^, ^3^ A positive bronchodilating test, which is indicated by significant FEV_1_ reversibility (a threshold of 12% and 200 ml reversibility to 400 μg salbutamol) combined with typical respiratory symptoms, is recommended by the Global Initiative for Asthma as proof of asthma. Airway hyper‐responsiveness (AHR) to MCT is reflective of another aspect of airway lability. A positive MCT with a provocative concentration of inhaled methacholine <16 mg/ml or ≤0.48 mg is highly sensitive for identifying AHR presence to allow asthma diagnosis in patients with typical symptoms suggestive of asthma and preserved baseline pulmonary function (FEV_1_ >70% pred).^3^, ^4^, ^5^ However, in most hospitals in China, performing an MCT costs >$73 and at least half an hour, which is expensive and time‐consuming. Moreover, it involves 11 steps,^6^ which is inconvenient for patients, technicians, and clinicians. Notably, MCT can cause unpleasant feelings in patients and can potentially induce severe bronchospasm.^6^ Therefore, the use of a pulmonary function test or a test combination to predict AHR presence or a positive MCT could provide great value to clinicians for asthma diagnosis.

Small airways, which are defined by an inner diameter <2 mm, represent a "quiet zone" and provide minimal resistance to airflow in normal adult lungs^7^; however, they are vulnerable to obstruction in asthma.^8^ In the diseased state, pulmonary function changes are not detected by standard spirometry until approximately 75% of the small airways are obstructed.^9^, ^10^ Therefore, it is crucial for predicting AHR presence in patients with a normal FEV_1_ for early stage asthma diagnosis based on small airway function parameters (SAFPs).^11^ Additionally, eosinophilic inflammation identified through fractional exhaled nitric oxide (FeNO) is also associated with the pathophysiological process of asthma.^12^ A previous study reported that the area under the curve (AUC) of the combination of forced expiratory flow (FEF) between 25% and 75% (FEF_25%–75%_) and FeNO for predicting AHR presence in patients with cough‐variant asthma was significantly higher than that of the parameters being applied separately (all *p* < 0.05); furthermore, FeNO >43 ppb and FEF_25%–75%_ <78.5% were the optimal cutoff values.^11^ Therefore, SAFPs combined with FeNO can improve the predictive value for AHR presence in asthma diagnosis.^11^ Further, compared with MCT, SAFP, and FeNO measurement are more accessible, safer, cheaper ($5–10 lower), and time‐saving (15–20 min).

Both SAFPs and FeNO are age dependent^13^, ^14^ with older age being an important small‐airway dysfunction (SAD) predictor in patients with asthma^9^, ^15^ and being associated with a greater daily dose of inhaled corticosteroid^9^ and a worse clinical asthma expression.^16^ Moreover, FeNO has been reported to decrease with aging in patients with asthma.^17^ Under‐ or overdiagnosis using these objective tests may occur when age is not considered.^18^ Therefore, age should be considered when using these parameters to predict a positive MCT for asthma diagnosis. A previous study defined older age as age ≥50 years.^9^ In our recent unpublished study on a Chinese population, 55 years was used as the optimal cut‐off value for determining the impact of age on small airway function (Lili Hou, unpublished paper). This study aimed to assess the impact of age on the predictive value of SAFPs, alone or combined with FeNO, for a positive MCT in patients with asthma‐suggestive symptoms and FEV_1_ ≥80% predicted. This could contribute to reduced over‐ or underprediction of a positive MCT for asthma diagnosis, as well as the elucidation of the treatment effect assessed by SAFPs and FeNO in patients with asthma with different age ranges.

## 2 METHODS

### 2.1 Patients and study design

This retrospective, cross‐sectional, multicentered, observational study recruited 846 adult patients with suspected asthma involving recurrent dyspnea, cough, chest tightness, and wheeze for ≥2 months. The recruited patients were referred to the Pulmonary Outpatient Clinic from January 2016 to September 2020. Among the recruited patients, a discovery cohort of 534 patients was enrolled from the electronic medical databases (EMD) of Shanghai General Hospital affiliated to Shanghai Jiao Tong University and Tongji Hospital Affiliated to Tongji University School of Medicine. Furthermore, a validation cohort of 312 patients was enrolled from the EMD of the Second Affiliated Hospital of Zhejiang University School of Medicine. These patients underwent standard spirometry, FeNO, and MCT. The participants in both cohorts were stratified by the previously mentioned cutoff age of 55 years to clarify the effect of age on the predictive value of SAFPs, alone or combined with FeNO, in patients with asthma‐suggestive symptoms and FEV_1_ ≥80% predicted. This study was approved by the ethics committee of the Institutional Review Board at Shanghai General Hospital (no. 2020 [30]). All the participants provided informed written consent for study participation.

### 2.2 Inclusion criteria

The inclusion criteria were as follows: age 18–80 years; history of chronic dyspnea, cough, and wheeze for ≥2 months; normal chest X‐ray or computed tomography results; predicted FEV_1_ of ≥80% with spirometric measurement, and having undergone MCT.

### 2.3 Exclusion criteria

The exclusion criteria were as follows: having fever or acute respiratory tract infections within the previous 8 weeks; having taken montelukast, long‐acting β_2_‐agonists, theophylline, anticholinergic agents, and inhaled or oral corticosteroids within the previous 4 weeks; or having comorbid severe systemic diseases, including chronic obstructive pulmonary diseases.

### 2.4 FeNO measurements

FeNO was measured using NIOX MINO (Aerocrine AB) at a standard flow rate of 50 ml/s following the American Thoracic Society (ATS)/European Respiratory Society (ERS) recommendations.^11^, ^19^ FeNO measurements were performed before the spirometric assessments and MCT since the involved breathing maneuvers could distort FeNO results.^11^

### 2.5 Spirometric measurements

Spirometry tests were performed between 8 and 11 a.m. using an MS‐PFT spirometer (Jaeger or SensorMedics). Spirometry tests followed the standards and recommendations of the ATS/ERS.^6^, ^20^ Expected values for lung function parameters were based on the prediction equation for patients in East China.

The following eight pulmonary function parameters were reviewed and analyzed: forced vital capacity (FVC), FEV_1_, FEV_1_/FVC (FEV_1_%), peak expiratory flow (PEF), FEF at 25% of FVC exhaled (FEF_25%_), FEF at 50% of FVC exhaled (FEF_50%_), FEF at 75% of FVC exhaled (FEF_75%_), and FEF between 25% and 75% (FEF_25%–75%_). Most of these parameters were presented as percentages of predicted values with FEV_1_/FVC being presented as the absolute value.

### 2.6 Methacholine challenge test

MCT was performed using the Jaeger APS Pro system using a Medic‐Aid sidestream nebulizer with doubling methacholine doses (0.0725–0.48 mg) following the ATS/ERS recommendations.^6^ FEV_1_ was measured at 3 min after each provocation step. Provocative doses that caused a 20% fall in FEV_1_ (PD_20_) was recorded; moreover, AHR was defined as positive if PD_20_ ≤ 0.48 mg.

Well‐trained technicians in each center performed FeNO, spirometry, and MCT measurements. An experienced clinician and technician discussed whether the MEFV curves met the ATS/ERS quality criteria for spirometry.^6^, ^20^

### 2.7 Outcomes

The primary outcome was the predictive value of SAFPs, alone or combined with FeNO, for the presence of a positive MCT in asthma diagnosis of the discovery cohort enrolled from two centers located in Shanghai, China. The secondary outcome was the confirmation of the primary outcome in a validation cohort enrolled from a third center located in Hangzhou, China.

### 2.8 Statistical analysis

Data were analyzed for normality of distribution using Kolmogorov–Smirov test. Normally distributed data were presented as mean ± *SD*. Nonnormally distributed data were expressed as median and interquartile range. Independent samples were compared using Student's *t* test (two‐tailed) or Mann–Whitney *U* test. Count data were presented as the percentage and between‐group comparisons were performed using the *χ* ^2^. Between‐parameter correlations were analyzed using Spearman's analysis.

For patients with suspected asthma who presented small airway dysfunction, MCT was considered the gold standard for defining AHR. Logistic regression was applied to determine the impact of continuous test variables with dichotomous state variables. Univariate logistic regression was used to determine the impact of the variables of interest; subsequently, multiple logistic regression was used to check whether the model could be improved. Predictive values of single or combined measurements were calculated by constructing receiver‐operating characteristic (ROC) curves and measuring AUCs.^11^ In the ROC plot, sensitivity was plotted against 100‐specificity. We determined the cut‐off value based on Youden's Index.

Analyses were performed using SPSS software package version 22.0 (IBM Corp.). ROC curve construction and AUCs comparisons were performed using MediCalc 19.0.4 software. AUCs were compared using the *χ* ^2^ test based on the method of Hanley and McNeil. Statistical significance was set at *p* < 0.05.

## 3 RESULTS

### 3.1 Baseline characteristics in the discovery cohort

The discovery cohort included 534 adults with symptoms of cough, chest tightness, dyspnea, and wheeze for ≥2 months. Among them, 276 (51.69%) patients exhibited a positive MCT. After stratification according to age, 291 and 243 participants were aged <55 and ≥55 years; among them, 148 (50.86%) and 128 (52.67%) participants had a positive MCT, respectively. Table 1 presents the baseline demographic and clinical characteristics of the patients. Based on the MCT results, there were no significant between‐group differences in age, sex, past smoking history, and BMI. Participants with positive MCT had lower PEF (all *p* < 0.05); however, the average value was within the normal range. Compared with the corresponding values in the negative MCT group, the FEF_25%_, FEF_50%_, FEF_75%_, FEF_25%–75%_ FEV_1_, and FEV_1_/FVC, values were significantly lower while the FeNO value was significantly higher (all *p* < 0.001, Table 1) in the positive MCT group. Moreover, the Spearman analysis revealed a weak correlation of PD20 with FEF_50%_ (*r* = 0.224, *p* < 0.001), FEF_25%–75%_ (*r* = 0.256, *p* < 0.001), and FeNO (*r* = −0.252, *p* < 0.001).

**TABLE 1:** Demographic data, spirometric parameters, and values for FeNO in patients with negative and positive methacholine challenge test in the discovery cohort

Characteristic variables	Negative MCT	Positive MCT	*p*
<55 years	*n*_1_ = 143	*n*_2_ = 148	
Male (*n*/%)	65 (45.45%)	60 (40.54%)	0.397
Age, years^a^	35.00 (30.00, 47.00)	40.00 (30.00, 48.00)	0.248
BMI, kg/m^2^ a	22.52 (19.95, 24.92)	22.31 (20.35, 24.64)	0.861
Past smoking history (*n*/%)	28 (19.58%)	31 (20.95%)	0.772
FEF_50%_, % predicted^a^	86.10 (68.60, 103.30)	65.50 (60.30, 73.65)	**<0.001**
FEF_25%_, % predicted^a^	101.10 (86.10, 115.60)	90.40 (75.45, 98.45)	**<0.001**
FEF_75%_, % predicted^a^	81.70 (61.20, 104.50)	69.80 (50.20, 86.28)	**<0.001**
FEF_25%–75%_, % predicted^a^	87.70 (73.80, 99.70)	68.35 (60.18, 73.35)	**<0.001**
PEF, % predicted^a^	103.60 (93.10, 114.60)	99.80 (90.30, 111.63)	**0.024**
FEV_1_, % predicted^a^	99.90 (94.60, 107.30)	96.60 (89.65, 103.15)	**<0.001**
FVC, % predicted^a^	101.60 (94.70, 107.10)	100.70 (93.20, 108.28)	0.966
FEV_1_/FVC, %^a^	85.23 (80.30, 91.65)	81.15 (77.61, 85.06)	**<0.001**
FeNO, ppb^a^	21.00 (13.00, 41.00)	53.00 (39.25, 93.00)	**<0.001**
≥55 years	*n*_1_ = 115	*n*_2_ = 128	
Male (*n*/%)	30 (26.09%)	34 (26.56%)	0.993
Age, years^a^	61.00 (56.00, 65.00)	61.00 (56.25, 64.00)	0.696
BMI, kg/m^2^ a	23.31 (21.67, 25.30)	23.62 (22.06, 25.74)	0.326
Past smoking history (n/%)	17 (14.78%)	14 (10.94%)	0.370
FEF_50%_, % predicted^a^	77.50 (65.30, 102.70)	60.90 (56.08, 65.48)	**<0.001**
FEF_25%_, % predicted^a^	101.00 (88.70, 117.60)	89.00 (76.30, 103.08)	**<0.001**
FEF_75%_, % predicted^a^	65.60 (49.10, 82.30)	53.45 (42.48, 70.78)	**<0.001**
FEF_25%–75%_, % predicted^a^	77.40 (63.70, 88.90)	59.55 (50.68, 69.83)	**<0.001**
PEF, % predicted^b^	109.20 ± 18.76	103.34 ± 18.87	**0.008**
FEV_1_, % predicted^a^	108.40 (98.50, 118.70)	97.85 (91.20, 107.98)	**<0.001**
FVC, % predicted^a^	110.90 (99.60, 122.30)	109.05 (100.85, 116.50)	0.130
FEV_1_/FVC, %^a^	81.23 (76.91, 85.84)	76.98 (73.97, 79.72)	**<0.001**
FeNO, ppb^a^	24.00 (16.00, 38.00)	49.00 (22.00, 56.75)	**<0.001**

*Note*: Bold font indicates statistical significance.

Abbreviations: BMI, body mass index; FEF25%, forced expiratory flow at 25% of forced vital capacity; FEF50%, forced expiratory flow at 50% of forced vital capacity; FEF75%, forced expiratory flow at 75% of forced vital capacity; FEF25%–75%, Forced expiratory flow between 25% and 75%; FeNO, fractional exhaled nitric oxide; FVC, forced vital capacity; FEV1, forced expiratory volume in 1 s; PEF, peak expiratory flow; MCT, methacholine challenge test.

^a^ median (IQR) values.

^b^ mean ± *SD* values.

### 3.2 Predictive values of single and combined variables for positive MCT

The predictive value of FEV_1_, FEV_1_/FVC, FEF_25%_, FEF_75%_, FEF_50%_, and FEF_25%–75%_, alone or combined with FeNO, was evaluated using ROC curves. Tables 2 and 3 show the sensitivity, specificity, PPV, NPV, and accuracy of each variable.

**TABLE 2:** Predictive values for predicting positive MCT in the discovery cohort

Characteristic Variables	AUC	Cut off values*	Sensitivity%	Specificity %	PPV %	NPV %	Accuracy%	+LR	−LR	Variable coef.	Constant coef.	*p*
All (*n* = 534)												
FEF_25%_, %predicted	0.681	99.7	75.36	53.10	63.2	66.8	59.74	1.61	0.46	−0.033	3.157	<0.001
FEF_50%_, %predicted	0.771	73.7	82.97	60.85	69.4	77.0	72.28	2.12	0.28	−0.059	4.426	<0.001
FEF_75%_, %predicted	0.635	68.1	60.14	60.08	61.7	58.5	60.11	1.51	0.66	−0.021	1.561	<0.001
FEF_25%–75%_, %predicted	0.774	75.2	85.51	65.12	72.4	80.8	75.66	2.45	0.22	−0.063	4.649	<0.001
FEV_1_, %predicted	0.652	97.9	53.62	68.60	64.6	58.0	60.86	1.71	0.68	−0.052	5.311	<0.001
FEV_1_/FVC, %	0.671	80.22	59.78	67.44	66.3	61.1	63.48	1.84	0.60	−0.066	5.551	<0.001
FeNO	0.754	43.0	61.23	84.11	80.5	67.0	77.28	3.85	0.46	0.039	−1.437	<0.001
≥55 years (*n* = 243)												
FEF_50%_, %predicted	0.794	65.0	73.44	77.39	78.3	72.4	75.31	3.25	0.34	−0.067	4.836	<0.001
FEF_25%–75%_, %predicted	0.769	62.9	65.62	77.39	76.4	66.9	71.19	2.90	0.44	−0.063	4.391	<0.001
FeNO	0.702	48.0	50.78	88.70	83.3	61.8	68.72	4.49	0.55	0.036	−1.151	<0.001
<55 years (*n* = 291)												
FEF_50%_, %predicted	0.760	74.2	76.35	67.83	71.1	73.5	72.16	2.37	0.35	−0.057	4.406	<0.001
FEF_25%–75%_, %predicted	0.792	74.9	83.78	74.13	77.0	81.5	79.04	3.24	0.22	−0.070	5.410	<0.001
FeNO	0.798	43.0	68.92	85.31	82.9	72.6	76.98	4.69	0.36	0.042	‐1.709	<0.001

*Note*: The cutoff values were selected by Youden Index.

Abbreviations: AUC, area under the curve; constant coef., constant coefficient of logistic regression; FEF_25%_, forced expiratory flow at 25% of forced vital capacity; FEF_50%_, forced expiratory flow at 50% of forced vital capacity; FEF_75%_, forced expiratory flow at 75% of forced vital capacity; FEF_25%–75%_: Forced expiratory flow between 25% and 75%; FEV_1_, forced expiratory volume in 1 s; FVC, forced vital capacity; FeNO, fractional exhaled nitric oxide; +LR, positive likelihood ratios; −LR, negative likelihood ratios; MCT, methacholine challenge test; NPV, negative predictive values; variable *p*, the *t* test of the characteristic variables coefficient for the *p*‐value; PPV, positive predictive values; variable coef., characteristic variables coefficient of logistic regression.

**TABLE 3:** Predictive values of small airway function parameters (FEF_50%_, FEF_25%–75%_) combined with FeNO in predicting positive MCT in the discovery cohort

Characteristic Variables	AUC	95% CI	Sensitivity %	Specificity %	PPV %	NPV %	Accuracy %	+LR	−LR	*p* ^a^	Variable coef.(*p*)	FeNO coef.(*p*)	Constant coef.
**All (*n* = 534)**													
FEF_50%_ + FeNO	0.858	0.826–0.887	78.26	79.46	80.3	77.4	78.65	3.81	0.27	<0.001	−0.063 (<0.001)	0.042 (<0.001)	3.076
FEF_25%–75%_ + FeNO	0.865	0.833–0.893	82.61	76.74	79.2	80.5	79.59	3.55	0.23	<0.001	−0.071 (<0.001)	0.046 (<0.001)	3.435
**≥55 years (*n* = 243)**													
FEF_50%_ + FeNO	0.851	0.800–0.893	80.47	75.65	78.6	77.7	78.19	3.30	0.26	0.002	−0.079 (<0.001)	0.047 (<0.001)	3.996
FEF_25%–75%_ + FeNO	0.844	0.792–0.887	85.94	71.30	76.9	82.0	78.60	2.99	0.20	0.001	−0.076 (<0.001)	0.048 (<0.001)	3.587
**<55 years (*n* = 291)**													
FEF_50%_ + FeNO	0.865	0.820–0.902	79.05	83.22	83.0	79.3	80.76	4.71	0.25	0.001	−0.052 (<0.001)	0.040 (<0.001)	2.374
FEF_25%–75%_ + FeNO	0.883	0.841–0.918	81.08	81.12	81.6	80.6	80.76	4.29	0.23	<0.001	−0.071 (<0.001)	0.045 (<0.001)	3.592

*Note*: The cutoff values were selected by the Youden Index. Bold font indicates statistical significance.

Abbreviations: AUC, area under the curve; constant coef., constant coefficient of logistic regression; FEF_50%_, forced expiratory flow at 50% of forced vital capacity; FEF_25%–75%_, forced expiratory flow between 25% and 75%; FeNO, fractional exhaled nitric oxide; FeNO coef. (*p*), FeNO coefficient of logistic regression and it's *t*‐test for the *p* value; +LR, positive likelihood ratios; −LR, negative likelihood ratios; MCT, methacholine challenge test; NPV, negative predictive values; PPV, positive predictive values; variable coef. (*p*), another characteristic variables coefficient of logistic regression and it's *t* test for the *p* value.

^a^ Compared with FEF_50%_ or FEF_25%–75%_ alone.

Before stratifying according to age, the AUCs of FEF_50%_ and FEF_25%–75%_ were 0.771 (95% confidence interval [CI]: 0.733–0.806) and 0.774 (95% CI: 0.736–0.809), respectively, which represented the two largest AUCs for a positive MCT diagnosis in the spirometry measurement (Table 2). The AUC of FeNO for a positive MCT diagnosis was 0.754 (95% CI: 0.716–0.790; Table 2).

**FIGURE 1:**
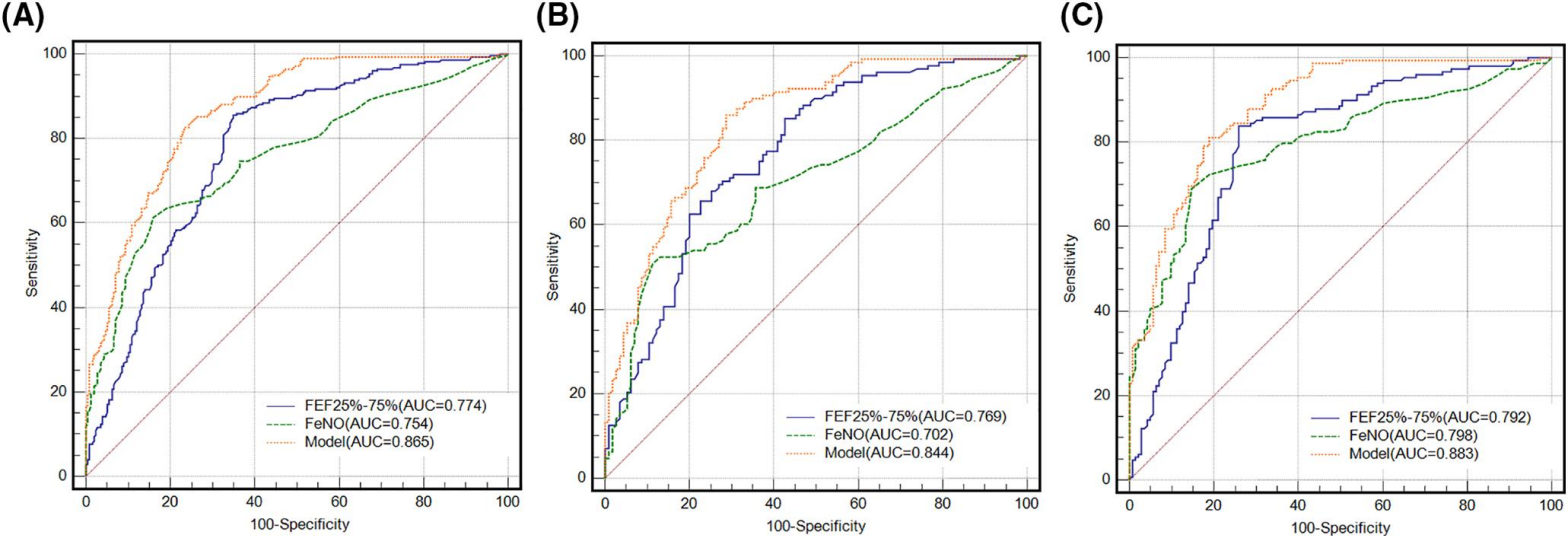
ROC curves for the model of FEF_25%–75%_ combined with FeNO in predicting positive bronchial provocation in patients of the discovery cohort prior to being stratified by age (A), patients aged ≥55 years (B), and patients aged <55 years (C). (A) *n* = 534, AUC_model_ = 0.865 (95% CI, 0.833–0.893); AUC_FEF25%–75%_ = 0.774 (95% CI, 0.736–0.809; *p* < 0.0001, compared with the model); AUC_FeNO_ = 0.754 (95% CI, 0.716–0.790; *p* = 0.5176 and <0.0001, compared with FEF_25%–75%_ alone and the model, respectively). (B) *n* = 243, AUC_model_ = 0.844 (95% CI, 0.792–0.887); AUC_FEF25%–75%_ = 0.769 (95% CI, 0.711–0.820; *p* = 0.0005, compared with the model); AUC_FeNO_ = 0.702 (95% CI, 0.640–0.759; *p* = 0.1700 and <0.0001, compared with FEF _25%–75%_ alone and the mode, respectively). (C) *n* = 291, AUC_model_ = 0.883 (95% CI, 0.841–0.918); AUC_FEF25%–75%_ = 0.792 (95% CI, 0.740–0.837; *p* < 0.0001, compared with the model); AUC_FeNO_ = 0.798 (95% CI, 0.747–0.842; *p* = 0.8724 and 0.0001, compared with FEF_25%–75%_ alone and the model, respectively)

To determine whether SAFPs combined with FeNO could improve prediction of a positive MCT, ROC analysis was performed using different combinations of SAFPs (FEF_50%_ and FEF_25%–75%_) with FeNO. The AUC for FEF_50%_ combined with FeNO was 0.858 (95% CI: 0.826–0.887), which was significantly higher than that of either FEF_50%_ (*p* < 0.0001) or FeNO alone (*p* < 0.0001). The AUC for FEF_25%–75%_ combined with FeNO was 0.865 (95% CI: 0.833–0.893), which was significantly higher than that of either FEF_25%–75%_ or FeNO alone (*p* < 0.001 for all; Table 3, Figure 1A).

In patients aged ≥55 years, the AUCs for FEF_50%_, FEF_25%–75%_, and FeNO were 0.794 (95% CI: 0.738–0.843), 0.769 (95% CI: 0.711–0.820), and 0.702 (95% CI: 0.640–0.759), respectively. The AUC for FEF_50%_ combined with FeNO was 0.851 (95% CI: 0.800–0.893), which was higher than that for either FEF_50%_ (*p* = 0.0119) or FeNO (*p* < 0.001) alone. The AUC for FEF_25%–75%_ combined with FeNO was 0.844 (95% CI: 0.792–0.887), which was significantly higher than that for either FEF_25%–75%_ or FeNO alone (all *p* < 0.001, Table 3, Figure 1B).

In patients aged <55 years, the AUC for FEF_50%_, FEF_25%–75%_, and FeNO was 0.760 (95% CI: 0.707–0.808), 0.792 (95% CI: 0.740–0.837), and 0.798 (95% CI: 0.747–0.842), respectively. The AUC for the combination of FEF_50%_ and FeNO was 0.865 (95% CI: 0.820–0.902), which was significantly higher than that for FEF_50%_ (*p* < 0.0001) or FeNO (*p* = 0.0005) alone. The AUC for FEF_25%–75%_ combined with FeNO was 0.883 (95% CI: 0.841–0.918), which was significantly higher than that for each of them (*p* < 0.0001 for FEF_25%–75%_ and *p* = 0.0001 for FeNO; Tables 2 and 3, Figure 1C).

### 3.3 Optimal cut‐off values for positive MCT prediction

The optimal cut‐off values were calculated based on Youden's Index. Prior to stratification according to age, the cut‐off values for FEF_50%_, FEF_25%–75%_, and FeNO were 73.7%, 75.2%, and 43 ppb, respectively. In patients aged ≥55 years, the cut‐off values for FEF_50%_, FEF_25%–75%_, and FeNO were 65.0%, 62.9%, and 48 ppb, respectively. On the other hand, in patients aged <55 years, the cut‐off values for FEF_50%_, FEF_25%–75%_, and FeNO were 74.2%, 74.9%, and 43 ppb, respectively (Table 2).

### 3.4 Predictive values of single and combined variables for positive AHR in the validation cohort

Table 4 shows the baseline demographic and pulmonary function characteristics of the validation cohort stratified according to age. In patients aged <55 years and ≥55 years, 63 (37.50%) and 57 out of 144 patients showed positive MCT, respectively.

**TABLE 4:** Demographic data, spirometric parameters, and values for FeNO in patients with negative and positive bronchial provocation tests in the validation cohort

Characteristic variables	Negative MCT	Positive MCT	*p* Value
**<55 years**	*n*_1_ = 105	*n*_2_ = 63	
**Male (*n*/%)**	53 (50.48%)	31 (49.21%)	0.873
**Age, years** a	34.00 (31.00, 41.00)	35.00 (29.00, 45.00)	0.743
**BMI, kg/m** ^**2**^ a	24.45 (21.41, 28.44)	23.11 (21.30, 25.39)	0.156
**Past smoking history (*n*/%)**	19 (18.10%)	19 (30.16%)	0.070
**FEF**_**50%**_ **, % predicted** a	97.00 (79.90, 115.35)	74.70 (64.80, 92.70)	**<0.001**
**FEF**_**25%**_ **, % predicted** b	101.30 ± 21.74	92.19 ± 15.37	**0.002**
**FEF**_**75%**_ **, % predicted** b	97.01 ± 27.71	75.24 ± 21.54	**<0.001**
**FEF**_**25‐75%**_ **, % predicted** a	94.10 (77.45, 111.50)	73.60 (61.04, 93.20)	**<0.001**
**PEF, % predicted** a	97.20 (86.85, 109.55)	90.40 (84.40, 100.20)	**0.015**
**FEV**_**1**_ **, % predicted** b	105.04 ± 11.48	98.73 ± 9.27	**<0.001**
**FVC, % predicted** b	102.58 ± 11.06	101.04 ± 8.53	0.312
**FEV**_**1**_ **/FVC, %** b	86.37 ± 5.48	82.36 ± 5.56	**<0.001**
**FeNO**, **ppb** a	22.00 (14.00, 38.50)	80.00 (28.00, 113.00)	**<0.001**
**≥55 years**	*n*_1_ = 87	*n*_2_ = 57	
**Male (*n*/%)**	30 (34.48%)	14 (24.56%)	0.206
**Age, years** a	62.00 (58.00, 67.00)	61.00 (59.00, 64.00)	0.352
**BMI, kg/m** ^**2**^ a	24.20 (22.27, 28.08)	24.03 (21.75, 25.85)	0.393
**Past smoking history (*n*/%)**	16 (18.39%)	8 (14.04%)	0.493
**FEF**_**50%**_ **, % predicted** a	78.00 (64.30, 98.8)	62.00 (57.90, 66.70)	**<0.001**
**FEF**_**25%**_ **, % predicted** b	98.38 ± 27.15	86.13 ± 14.34	**0.001**
**FEF**_**75%**_ **, % predicted** a	94.30 (73.40, 113.90)	62.00 (57.90, 66.70)	**<0.001**
**FEF**_**25‐75%**_ **, % predicted** a	79.40 (64.10, 101.30)	62.80 (56.70, 66.00)	**<0.001**
**PEF, % predicted** a	96.80 (84.30, 109.20)	87.00 (79.45, 100.20)	**0.011**
**FEV**_**1**_ **, % predicted** a	106.60 (97.40, 115.10)	94.70 (89.80, 106.40)	**<0.001**
**FVC, % predicted** a	100.80 (90.50, 111.90)	99.30 (90.20, 107.50)	0.491
**FEV**_**1**_ **/FVC, %** b	85.44 ± 4.88	81.46 ± 5.70	**0.001**
**FeNO**, **ppb** a	24.00 (15.00, 44.00)	53.00 (29.50, 82.00)	**<0.001**

*Note*: Bold font indicates statistical significance.

Abbreviations: BMI, body mass index; FEF_25%_, forced expiratory flow at 25% of forced vital capacity; FEF_50%_, forced expiratory flow at 50% of forced vital capacity; FEF_75%_, forced expiratory flow at 75% of forced vital capacity; FEF_25%–75%_, Forced expiratory flow between 25% and 75%; FeNO, fractional exhaled nitric oxide; FVC, forced vital capacity; FEV_1_, forced expiratory volume in 1 s; PEF, peak expiratory flow.

^a^ median (IQR) values.

^b^ mean ± *SD* values.

**FIGURE 2:**
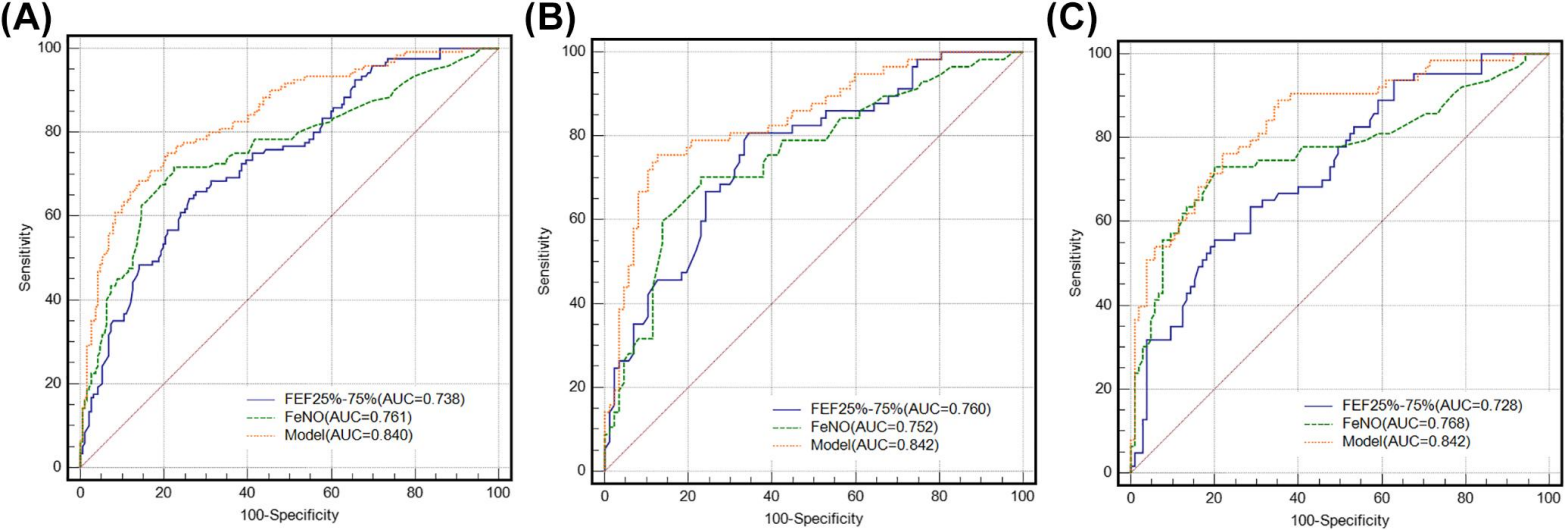
ROC curves for the model of FEF_25%–75%_ combined with FeNO in predicting positive bronchial provocation in the patients of validation cohort prior to being stratified by age (A), patients aged ≥ 55 years (B) and patients aged < 55 years (C). (A) *n* = 312, AUC_model_ = 0.840 (95% CI, 0.795–0.879); AUC_FEF25%–75%_ = 0.738 (95% CI, 0.686–0.786; *p* < 0.0001, compared with the model); AUC_FeNO_ = 0.761 (95% CI, 0.710–0.807; *p* = 0.5812 and 0.0006, compared with FEF _25%–75%_ alone and the model, respectively). (B) *n* = 144, AUCmodel = 0.842 (95% CI, 0.772–0.898); AUC_FEF25%–75%_ = 0.760 (95% CI, 0.682–0.827; *p* = 0.0084, compared with the model); AUC_FeNO_ = 0.752 (95% CI, 0.674–0.821; *p* = 0.9018 and 0.0171, compared with FEF_25%–75%_ alone and the model, respectively). (C) *n* = 168, AUC_model_ = 0.842 (95% CI, 0.778–0.894); AUC_FEF25%–75%_ = 0.728 (95% CI, 0.655–0.794; *p* = 0.0007, compared with the model); AUC_FeNO_ = 0.768 (95% CI, 0.697–0.830; *p* = 0.4900, and 0.0149, compared with FEF_25%–75%_ alone and the model, respectively)

Prior to stratification according to age, the AUCs for FEF_50%_, FEF_25%–75%_, and FeNO were 0.737 (95% CI: 0.684–0.785), 0.738 (95% CI: 0.686–0.786), and 0.761 (95% CI: 0.710–0.807) respectively. The AUC for FEF_50%_ combined with FeNO was 0.842 (95% CI: 0.797–0.881), which was significantly higher than that for FEF_50%_ (*p* < 0.0001) or FeNO alone (*p* = 0.0003). The AUC for FEF_25%–75%_ combined with FeNO was 0.840 (95% CI: 0.795–0.879), which was significantly higher than that for either FEF_25%–75%_ (*p* < 0.0001) or FeNO used alone (*p* = 0.0006; Tables 5 and 6, Figure 2A).

**TABLE 5:** Predictive values for predicting positive MCT in the validation cohort

Characteristic Variables	AUC	Cut off values	Sensitivity%	Specificity%	PPV%	NPV%	Accuracy%	+LR	−LR	Variable coef.	Constant coef.	*p*
**All (*n* = 312)**												
FEF_50%_, %predicted	0.737	75.8	68.33	69.27	58.2	77.8	68.91	2.22	0.46	−0.041	2.878	<0.001
FEF_25%–75%_, %predicted	0.738	74.0	65.83	72.40	59.8	77.2	69.87	2.38	0.47	−0.043	2.971	<0.001
FeNO	0.761	40.0	71.67	77.60	66.7	81.4	75.32	3.20	0.37	0.028	−1.795	<0.001
**≥55 years (*n* = 144)**												
FEF50%, %predicted	0.775	67.7	80.70	66.67	61.3	84.1	72.22	2.42	0.29	−0.056	3.657	<0.001
FEF25%–75%, %predicted	0.760	66.7	80.70	65.52	60.5	83.8	71.53	2.34	0.29	−0.054	3.517	<0.001
FeNO	0.752	47.0	70.18	77.01	66.7	79.8	74.31	3.05	0.39	0.028	−1.654	<0.001
**<55 years (*n* = 168)**												
FEF_50%,_ %predicted	0.727	75.8	55.56	80.95	63.6	75.2	71.43	2.92	0.55	−0.041	3.080	<0.001
FEF_25%–75%_, %predicted	0.728	75.4	55.56	80.00	62.5	75.0	70.83	2.78	0.56	−0.042	3.075	<0.001
FeNO	0.768	40.0	73.02	80.00	68.7	83.2	77.38	3.65	0.34	0.028	−1.958	<0.001

*Note*: The cutoff values were selected by the Youden Index.

Abbreviations: AUC, area under the curve; constant coef., constant coefficient of logistic regression; FEF_50%_, forced expiratory flow at 50% of forced vital capacity; FEF_25%–75%_, Forced expiratory flow between 25% and 75%; FeNO, fractional exhaled nitric oxide; +LR, positive likelihood ratios; −LR, negative likelihood ratios; MCT, methacholine challenge test; NPV, negative predictive values; *p*, the *t* test of the characteristic variables coefficient for the *p* value; PPV, positive predictive values; variable coef., characteristic variables coefficient of logistic regression.

**TABLE 6:** Predictive values of small airway function parameters (FEF_50%_, FEF_25%–75%_) combined with FeNO in predicting positive MCT in the validation cohort

Characteristic variables	AUC	95% CI	Sensitivity %	Specificity%	PPV %	NPV %	Accuracy%	+LR	−LR	*p* ^a^	Variable coef.(*p*)	FeNO coef.(*p*)	Constant coef.
**All (*n* = 312)**													
FEF_50%_ + FeNO	0.842	0.797–0.881	64.17	90.62	81.1	80.2	80.45	6.84	0.40	<0.001	−0.046 (<0.001)	0.029 (<0.001)	1.879
FEF_25%–75%_ + FeNO	0.840	0.795–0.879	68.33	85.94	75.2	81.3	79.17	4.86	0.37	0.001	−0.046 (<0.001)	0.029 (<0.001)	1.822
**≥55 years (*n* = 144)**													
FEF_50%_ + FeNO	0.851	0.782–0.905	73.68	89.66	82.4	83.9	83.33	7.12	0.29	0.021	−0.068 (<0.001)	0.033 (<0.001)	3.145
FEF_25%–75%_ + FeNO	0.842	0.772–0.898	75.44	87.36	79.6	84.4	81.94	5.97	0.28	0.017	−0.059 (<0.001)	0.030 (<0.001)	2.618
**<55 years (*n* = 168)**													
FEF_50%_ + FeNO	0.842	0.777–0.893	84.13	71.43	63.9	88.2	76.19	2.94	0.22	0.011	−0.039 (<0.001)	0.027 (<0.001)	1.525 (<0.001)
FEF_25%–75%_ + FeNO	0.842	0.778–0.894	76.19	78.10	67.6	84.5	77.38	3.48	0.30	0.015	−0.040 (<0.001)	0.027 (<0.001)	1.490

*Note*: The cutoff values were selected by the Youden Index. Bold font indicates statistical significance.

Abbreviations: AUC, area under the curve; constant coef., constant coefficient of logistic regression; FEF_50%_, forced expiratory flow at 50% of forced vital capacity; FEF_25%–75%_, Forced expiratory flow between 25% and 75%; FeNO, fractional exhaled nitric oxide; FeNO coef. (*p*), FeNO coefficient of logistic regression and it's *t* test for the *p* value; +LR, positive likelihood ratios; −LR, negative likelihood ratios; MCT, methacholine challenge test; NPV, negative predictive values; PPV, positive predictive values; variable coef. (*p*), another characteristic variables coefficient of logistic regression and it's *t* test for the *p* value.

^a^ Compared with FEF_50%_ or FEF_25%–75%_ alone.

In patients aged ≥55 years, the AUCs for FEF_50%_, FEF_25%–75%_, and FeNO were 0.775 (95% CI: 0.689–0.840), 0.760 (95% CI: 0.682–0.827), and 0.752 (95% CI: 0.674–0.830), respectively. The AUC for FEF_50%_ combined with FeNO was 0.851 (95% CI: 0.782–0.905), which was significantly higher than each of them used individually (both *p* < 0.05). The AUC for FEF_25%–75%_ combined with FeNO was 0.842 (95% CI: 0.772–0.898), which was significantly higher than that for either FEF_25%–75%_ (*p* = 0.0084) or FeNO (*p* = 0.0171) used alone (Tables 5 and 6, Figure 2B).

In patients aged <55 years, the AUCs for FEF_50%_, FEF_25%–75%_, and FeNO were 0.727 (95% CI: 0.653–0.793), 0.728 (95% CI: 0.655–0.794), and 0.768 (95% CI: 0.697–0.830), respectively. The AUC for FEF_50%_ combined with FeNO was 0.842 (95% CI: 0.777–0.893), which was significantly higher than that for FEF_50%_ (*p* = 0.0007) or FeNO (*p* = 0.01137) used alone. The AUC for FEF_25%–75%_ combined with FeNO was 0.842 (95% CI: 0.778–0.894), which was higher than each of them used singly (*p* = 0.0007 for FEF_25%–75%_ and *p* = 0.0149 for FeNO; Tables 5 and 6, Figure 2C).

Before stratifying according to age, the cut‐off values for FEF_50%_, FEF_25%–75%_, and FeNO were 75.8%, 74.0%, and 40 ppb, respectively. In patients aged ≥55 years, the cut‐off values for FEF_50%_, FEF_25%–75%_, and FeNO were 67.7%, 66.7%, and 47 ppb, respectively. In patients aged <55 years, the cut‐off values for FEF_50%_, FEF_25%–75%_, and FeNO were 75.8%, 75.4%, and 40 ppb, respectively (Tables 5 and 6).

## 4 DISCUSSION

This study aimed to determine the age effect on the predictive utility of SAFPs, alone or combined with FeNO, for a positive MCT in patients in two age groups (54 years and under, 55 years and over) with asthma‐suggestive symptoms and FEV_1_ ≥ 80% predicted. The main findings of this study are as follows: compared with the corresponding values in the negative MCT group, in the positive MCT group, the FEF_50%_ and FEF_25%–75%_ values were significantly lower while the FeNO value was higher; compared with participants aged ≥55 years, patients aged <55 years had a higher optimal cutoff value of SAFPs (FEF_25%–75%_ and FEF_50%_), a lower optimal cutoff value of FeNO and a higher AUCs for the combination of SAFPs and FeNO (>0.86); and the predictive value of SAFPs combined with FeNO in both age groups for a positive MCT diagnosis was significantly improved in patients with asthma‐suggestive symptoms and FEV_1_ ≥ 80% predicted.

In patients with asthma‐suggestive symptoms and normal or near‐normal pulmonary functions values, MCT is appropriate for confirming or excluding an asthma diagnosis.^6^ However, MCT is expensive, time‐consuming, and inconvenient. Therefore, there is a need for cheaper, safer, and simpler tests for predicting a positive MCT, especially in hospitals lacking access to MCT. Previous studies have reported that SAFPs combined with FeNO can predict AHR presence in patients with cough‐variant asthma,^11^ as well as patients with asthma‐suggestive symptoms and a normal FEV_1_ (Min Zhang et al., unpublished paper). This combination was confirmed to be easier, safer, cheaper, and time‐saving than MCT. However, these studies did not address the age effect on the predictive value of these parameters.

In our study, the AUCs of FEF_50%_ and FEF_25%–75%_ for predicting AHR in the discovery cohort did not significantly differ in both age groups (Table 2). This is consistent with previous findings that these two SAFPs had similar predictive values and good correlation for predicting a positive MCT for asthma diagnosis.^11^, ^21^

The current study showed that FEF_25%–75%_ and FEF_50%_ values in patients with a positive MCT were <80% predicted were much lower than those in patients with a negative MCT independent of age stratification. This indicated that SAD was present in early stage asthma. The FEF_50%_ and FEF_25%–75%_ values were lower in patients aged ≥55 years with a positive MCT than in those aged <55 years (all *p* < 0.01, data not shown). This is consistent with previous results that normal aging contributed to SAD in different subgroups of patients with asthma^9^, ^22^, ^23^; moreover, it indicates that SAD might be more severe and common in older patients than in younger patients. Consistent with this finding, in our study, the cut‐off value of FEF_25%–75%_ and FEF_50%_ for a positive MCT prediction for asthma diagnosis in patients aged ≥55 years (65.0% for FEF_25%–75%_ and 62.9% for FEF_50%_) was much lower than that in patients aged <55 years (74.2% for FEF_25%–75%_ and 74.9% for FEF_50%_), which is consistent with previous findings that the optimal cut‐off value of FEF_25%–75%_ was lower in Chinese adults with CVA (78.5%)^11^ than in Chinese children with CVA (80.5%).^24^ Moreover, the cut‐off values of FEF_25%–75%_ and FEF_50%_ in older patients with a positive MCT (65.0% for FEF_25%–75%_ and 62.9% for FEF_50%_) decreased significantly compared with those in patients not stratified by age (75.2% for FEF_25%–75%_ and 73.7% for FEF_50%_). This is indicative of an overdiagnosis in older patients with asthma if not stratified by age when using FEF_25%–75%_ and FEF_50%_ to predict a positive MCT in patients with suspected asthma, which supports the previous finding that age‐specific reference values should be considered for asthma diagnosis.^18^ Compared with patients aged <55 years with a positive MCT, patients aged ≥55 years had lower FVC, FEV_1_, and FEV_1_/FVC, although it was within the normal range (all *p* < 0.05). There were no significant between‐age‐group differences in the FEF_25%_ and PEF (all *p* > 0.05) in patients with a positive MCT. Furthermore, we found that the predictive value of FEF_25%–75%_ was lower in elder patients (Table 2), which was consistent with previous findings (0.800 for children and 0.702 for adults with CVA, respectively).^11^, ^24^ Notably, regardless of age stratification, the FEF_25%–75%_ and FEF_50%_ alone could not predict AHR in patients with suspected asthma since the AUC was <0.8. Interestingly, in our study, the predictive value of SAFPs (FEF_25%–75%_ and FEF_50%_) combined with FeNO for AHR diagnosis was significantly improved in both age groups.

FeNO is widely used as a noninvasive biomarker for monitoring airway eosinophilic inflammation and predicting corticosteroid sensitivity in allergic diseases such as asthma.^25^ Specifically, FeNO is currently helpful for ruling out asthma. With 43 ppb as the optimal cut‐off value, Bao et al.^11^ reported that the sensitivity, specificity, PPV, and NPV were 71.59%, 82.02%, 66.30%, and 85.40% respectively, in clinical AHR prediction for Chinese patients with CVA, which indicated that FeNO was valuable as a negative predictive parameter for discriminating patients with AHR. Schleich et al.^26^ reported that for patients with suspected asthma, FeNO >34 ppb had a relatively low predictive value (AUC = 0.62) for AHR diagnosis. Before age stratification, we found that FeNO >43 ppb has a sensitivity, specificity, PPV, and NPV of 61.23%, 84.11%, 80.50%, and 67.0%, respectively, for predicting a positive MCT in patients with suspected asthma. The FeNO value has been reported to be affected by age.^13^, ^14^ Specifically, FeNO >25.5 ppb had a high predictive value (AUC = 0.905) with a sensitivity and specificity of 82.2% and 90.0%, respectively, for CVA diagnosis in Chinese children with an average age of 8 years,^24^ which differed from that in the aforementioned Chinese adults with CVA.^11^ Consistent with this trend, our study showed that the FeNO value was higher in patients with positive AHR aged <55 years than in those aged ≥55 years (*p* < 0.05), which indicated that airway eosinophilic inflammation might be more severe in younger patients with asthma who may be more sensitive to corticosteroids. This is consistent with previous findings that older patients with asthma had lower FeNO level, which indirectly indicates predominant neutrophilic bronchial inflammation in elderly patients that contributes to greater airflow limitation.^17^ However, our findings are inconsistent with a previous report that FeNO level increased with aging.^27^ However, this previous study was conducted in a healthy population without a smoking history.^27^ Compared with aging, bronchial inflammation might be more crucially involved in affecting the FeNO level in the present study. Before age stratification, the cut‐off value of FeNO for predicting AHR was 43 ppb; however, for patients aged ≥55 years, this value was 48 ppb, which indicated an overdiagnosis in older participants. In patients aged ≥55 years, FeNO >48 ppb had a high specificity (88.70%), but a relative low sensitivity (50.78%), for identifying patients with asthma, which indicated that FeNO is limited as a sole diagnostic test for these patients. In patients aged <55 years, FeNO >43 ppb had a relative low sensitivity 68.92% and NPV 72.60%, but a higher specificity 85.31% and PPV 82.90%, for predicting AHR in patients with typical asthma‐like symptoms and an asthma‐suggestive history.

The AUC for FEF_50%_ (FEF_25%–75%_) combined with FeNO was 0.858 (0.865), 0.865 (0.883), and 0.851 (0.844) in patients without age stratification, patients aged <55 years, and patients aged ≥55 years, respectively, which was significantly higher than that for either FEF_50%_ (FEF_25%–75%_) or FeNO used alone. These findings suggest that FEF_50%_ (FEF_25%–75%_) combined with FeNO could improve the predictive value for AHR diagnosis, which is consistent with a previous study on patients with CVA.^11^ In patients aged ≥55 years, compared with the corresponding values calculated from SAFPs used alone, SAFPs combined with FeNO had a higher specificity (80.47% for FEF_50%_, 85.94% for FEF_25%–75%_) and negative predictive value (77.70% for FEF_50%_, 82.00% for FEF_25%–75%_); in patients aged <55 years, the corresponding values of specificity (83.22% for FEF_50%_, 81.12% for FEF_25%–75%_) and positive predictive value (83.00% for FEF_50%_, 81.60% for FEF_25%–75%_) also improved when SAFPs were combined with FeNO. This indicated that the combined use of optimal cut‐off values of SAFPs and FeNO contributed to improved prediction for a positive MCT detection in patients of both age groups with suspected asthma with FEV_1_ ≥80% predicted.

Finally, we validate the findings from the discovery cohort using a validation cohort. This indicates that our findings could be generalized in patients with asthma in other care centers in China.

This study has several limitations. First, SAD included airway wall thickening, airway narrowing, and air trapping, and so forth.^28^ However, we did not further confirm these changes using computed tomography or tissues. Further studies should elucidate the associations among changes of pulmonary function and imaging and histological structures of peripheral airways. Second, FeNO was specifically correlated with airway eosinophilic, but not neutrophilic, inflammation. In previous studies, the diagnostic accuracy of FeNO increased upon exclusion of patients with neutrophilic inflammation.^29^, ^30^ There is a need for sputum eosinophil and neutrophil measurements to confirm asthma phenotypes. Third, comorbid diseases, including rhinitis, gastroesophageal reflux disease, and so forth, may affect result interpretation, which should have been considered in this study. However, given the retrospective and cross‐sectional design of this study, there was limited information regarding these comorbid diseases. Furthermore, the predictive value of SAFPs, alone or combined with FeNO, for the presence of a positive MCT was only assessed in two age groups (≤54 years and ≥55 years). However, asthma can occur at any age^31^, ^32^ and exhibits a similar rate among adults.^33^ Moreover, small airway function has been shown to decline with aging^22^, ^23^, ^34^ and contribute to asthma emergence and exacerbation.^23^, ^35^ Unfortunately, there is limited information regarding the starting age of decline for small airway function in Chinese healthy individuals and patients with asthma. Therefore, the trend of the value for predicting a positive MCT in these patients should be evaluated in more different age groups. Unfortunately, some groups stratified at 10‐year intervals in this study had few patients with asthma; therefore, future studies should recruit a larger study group to better represent the population.

## 5 CONCLUSIONS

Patients with asthma with SAD, which was affected by aging, were more prone to have a positive MCT. Therefore, Chinese patients with FEV_1_ ≥ 80% and an asthma‐suggestive history should be stratified by age (55 years) when SAFPs (FEF_50%_, FEF_25%–75%_), alone or combined with FeNO, are used to predict the presence of a positive MCT in asthma diagnosis.

## CONFLICT OF INTERESTS

The authors declare that they have no competing interests.

## AUTHOR CONTRIBUTIONS

Lili Hou, Lei Zhu, and Min Zhang were responsible for conceptualization, design of the study, and drafting the manuscript. Min Zhang and Lei Zhu was responsible for funding acquisition. Lili Hou, Gang Huang, Huahao Shen, and Li Yu were responsible for data collection and interpretation. Lili Hou, Huijuan Hao, and Huahao Shen were responsible for data analysis and interpretation. All authors reviewed the article critically for important intellectual content and approved the final version to be submitted.
